# A global approach to safety assessment of medicinal plants

**DOI:** 10.2471/BLT.24.292879

**Published:** 2025-09-29

**Authors:** Bhushan Patwardhan, Sarika Chaturvedi, Roy Upton, Jennifer Hunter, L Susan Wieland, Nessma El-Nabawy, Anchalee Chuthaputti, Chunyu Wei, Kofi Donkor, Kim Sungchol, Evelyn Wolfram, Geetha Krishnan Gopalakrishna Pillai, Shyama Kuruvilla

**Affiliations:** a Centre for Complementary and Integrative Health, Interdisciplinary School of Health Sciences, Savitribai Phule Pune University, Ganeshkhind, Pune, 411007 India.; b Dr. D.Y. Patil Medical College Hospital and Research Centre, Dr D Y Patil Vidyapeeth, Pune, India.; c American Herbal Pharmacopoeia, Soquel, United States of America (USA).; d Health Research Group Pty Ltd, Sydney, Australia.; e Center for Integrative Medicine, University of Maryland, College Park, USA.; f General Administration for Registration of Herbal Products, Egyptian Drug Authority, Cairo, Egypt.; g Department of Thai Traditional and Complementary Medicine, Ministry of Public Health, Nonthaburi, Thailand.; h China Academy of Chinese Medicine Sciences, Beijing, China.; i Department of Pharmacology/Toxicology, Centre for Plant Medicine Research, Mampong-Akuapem, Ghana.; j Traditional Complementary Integrative Unit, World Health Organization, Geneva, Switzerland.; k Zurich University of Applied Sciences Wadenswil, Zurich, Switzerland.; l Global Traditional Medicine Centre, World Health Organization, Jamnagar, India.; Correspondence to Bhushan Patwardhan (email: bpatwardhan@gmail.com).

Establishing a robust global framework for assessing the safety of plants used for medicinal purposes (medicinal plants) is complex. Unlike synthetic drugs, plant-based products often lack standardized codification, quantification or context-specific usage data. Medicinal plant products are classified differently worldwide: in some countries (such as in the United States of America), they are treated as foods or dietary supplements unless manufacturers make therapeutic claims; in other jurisdictions, geographic areas and countries (such as in the European Union, the Asian continent, Australia or Canada), they are regulated as low-risk medicines.^1^ Different jurisdictions vary widely in cultural acceptance or bias towards medicinal plant use. This fragmented landscape results in inconsistent safety standards, reporting protocols and access to markets for practitioners, patients and consumers.

For centuries, plants have been used in diverse cultural traditions worldwide for health promotion and disease management, and as foundations for biomedical drug development. Use of medicinal plants continues to grow globally, both in traditional medical systems and in commercial trade.^2^ This expanded use has outpaced the development of harmonized frameworks for evaluating medicinal plant safety, especially when products are marketed beyond their traditional contexts, prepared in novel ways or used concomitantly with pharmaceuticals. Furthermore, newer tools and techniques such as chemoinformatics, toxicogenomics, network pharmacology, ingredient analysis and artificial intelligence (AI) applied judiciously can strengthen medicinal plant safety assessment.

Here, we outline key challenges and propose considerations to advance a globally applicable, unbiased safety framework for medicinal plants.

## Terminologies

Medicinal plants refer to botanical ingredients used for health-related purposes, including raw, processed and finished products.^3^ Traditional use refers to the experiential and empirically established use of such plants over generations within specific cultural and medical systems. Novel use describes preparations made from medicinal plants that may be used outside of their culturally and ecologically defined contexts. The safety and efficacy of medicinal plants are shaped by cultural and ecological contexts, including species and plant parts used, cultivation and harvesting practices, preparation methods, dosage, indications, co-administered substances, soil, climate, local biodiversity and associated traditional knowledge. All these determinants underscore the importance of terroir, that is, the unique environmental factors, farming practices and plants’ specific growth habitat, in the use and safety considerations of medicinal plants.

Failure to explicitly distinguish between medicinal plant products used in an ecologically and culturally informed manner and commercial products traded globally contributes to misinformation and, at times, misguided policy and regulatory responses.^4^

## Historical presumption of safety

The primary basis for permitting human use of medicinal plants remains their apparent historical safety, provided that no contradictory modern data exist.^5^ Most medicinal plants, like conventional foods (that is, foods produced through agricultural practices including the use of pesticides and chemicals), remain in the public domain owing to cultural acceptance of their perceived safety and benefits, despite lacking formal toxicology data. Contrastingly, modern drugs being novel, their safety cannot be presumed, and are hence subjected to formal toxicological studies before market authorization.

In contrast to traditional herbal therapies, biomedical drug discovery abandoned whole plant medicine in favour of isolated chemical compounds. While the earliest compounds were derived from plants, this changed with the advent of synthetic chemistry^6^ that led to the introduction of isolated chemicals to which humans had not been exposed. The shift from plants to isolated chemicals necessitated formal pre-clinical toxicological studies to ensure drug safety before use. With few exceptions, this approach to safety assessment frequently extends to medicinal plant safety assessment, despite generations of continued use and knowledge establishing relative safety for their traditional use. In cultures that have continuously maintained a strong medicinal plant tradition (such as China and India), the concept of investigating isolated plant chemicals is perceived as a foreign paradigm that is inconsistent with traditional practices.

Additionally, the mere presence of a compound of toxicological concern in a plant does not equate to functional toxicity.^7^ Toxicity of plants is not determined solely by toxic compounds but by factors such as bioavailability, dose, phytochemical interactions, metabolism, environmental conditions, traditional detox practices and human physiological defences.^8^ Therefore, safety assessments must be based on information about traditional use and not only on the potential for toxicity based on theoretical concerns, isolated constituents or preclinical experiments that may be inconsistent with traditional use. These principles are central to rational safety policies for medicinal plants. Modern analytics improve the detection of cumulative toxicity, advancing assessments beyond historical limitations.

## Complexity of medicinal plants

Plants contain a multitude of bioactive compounds. This chemo-diversity makes plants less suited for assessment using conventional research paradigms focused on drug discovery through molecules, targets and pathways. Chemo-diversity can pose challenges to standardization of medicinal plant formulations, but it can also offer therapeutic advantages. Additionally, cultivation practices, harvesting methods and environmental factors can influence plant chemistry, impacting both safety and therapeutic outcomes. In some cases, chemo-diversity results in a greater degree of safety and efficacy than the use of isolated compounds.^9^ Safety assessments disregarding this understanding can lead to inappropriate conclusions and decisions.^9^ Safety assessments should hence be informed by traditional knowledge and not rely exclusively on models developed for commercial drug discovery. When a plant has a well-established history of safe traditional use, applying safety assessment models designed for new commercial drug compounds may be unnecessary and of little value. However, such assessments can be essential if the way the plant is used changes or if it is introduced into new contexts.

## Conventional safety assessment 

In conventional health-care systems, safety assessments of medicinal plants rely heavily on preclinical toxicological studies and, to a lesser degree, clinical trials. While valuable, these models are often not designed to emulate traditional use, which is typically individualized to patients, adapted to multiple morbidities and adjusted over the course of treatment. Clinical trials to determine safe dosage of medications are best suited to assessing standardized, single-component therapies, and hence do not suit complex entities such as plants in real-world use. Therefore, researchers are increasingly exploring alternatives like pragmatic trials that test effectiveness in real-world settings and provide evidence directly applicable to practice. Short-term trials of interventions (plant or synthetic) may also not detect rare or long-term adverse events, such as hepatotoxicity or genotoxicity. Notably, traditional practices sometimes limit the use of particular botanical ingredients due to concerns of toxicity. Therefore, short-term clinical trials assessing the use of such plants may not reflect the entirety of safe traditional practices. On the other hand, clinical trials may address the current gap in evidence on the concurrent use of medicinal plants with conventional medications. Conventional safety assessments also often overlook historical and cultural usage data, underutilize real-world evidence and pharmacovigilance, and fail to address polyherbal formulations. Additionally, the interaction potential of concomitant medicinal plant–pharmaceutical use is one of the areas that need investigation to identify both negative and positive interactions.^4^ However, appropriate models for assessing medicinal plant–drug interactions are lacking.

## Real-world evidence

Traditional use, practitioner insights and patient outcomes form real-world evidence that can complement preclinical and clinical data in guiding safe medicinal plant use. Real-world evidence of traditional knowledge systems offers insights into patient-specific factors, population-level safety signals, usage patterns, long-term effects, risk-management strategies and informed medicinal plant use based on a strong foundation of didactic education coupled with generations of accumulated and recorded human experience. Investigators and regulators need to assess all available evidence, from clinical trials to traditional data to ensure safety assessments and related decisions are accurate and contextually relevant. They must also systematically collect real-world evidence linked to specific interventions, contextualized within the health system, and subjected to critical appraisal to make informed policy decisions.^10^ Safeguarding related traditional knowledge, including appropriate attribution and protection of intellectual property, is essential to ensure ethical integration and benefit-sharing.

## Comprehensive safety assessment

All evidence types have strengths and limitations. Here, we advocate for a multifaceted, contextual safety assessment model that synthesizes the full spectrum of evidence on medicinal plant safety. A comprehensive framework should include codified traditionally informed usage data (indications, dosage, duration and contraindications); appropriate characterization of specific products or classes of products; preclinical and clinical safety data when available; pharmacovigilance and post-marketing surveillance; and digital tools (such as AI and databases) for pattern recognition and early signal detection, and for the integration of ethnobotanical and genomic data to gain novel insights.

## Global safety system

We recommend the establishment of a global hub for safety assessment of medicinal plants, ideally under the guidance of the World Health Organization, that can serve as an unbiased knowledge-sharing and policy advisory platform where international databases on all aspects of medicinal plant safety, including knowledge from traditional medicine systems are developed and maintained. The hub would also promote regulatory convergence while respecting regional traditions, and support countries in enhancing pharmacovigilance for medicinal plants. Through the hub, innovations and technologies that allow for the collection and critical review of real-world and research-derived evidence would be developed and promoted. The hub would moreover support the development of systematic reviews and medicinal plant safety assessment reports informed by all evidence in conjunction with expert oversight, and assess implications of therapeutic product quality, access, affordability and acceptability on their role in achieving universal health coverage (UHC). Finally, the hub would anchor the work within global frameworks for intellectual property rights, equitable benefit-sharing, and biodiversity conservation, such as the World Intellectual Property Organization Treaty and Nagoya Protocol.^11^

Ensuring safe use of medicinal plants amid rising global trade is a public health priority. Doing so requires balanced evaluation of diverse knowledge systems and evidence sources to guide sound policy decisions. Moving forward, stronger global collaboration and modern tools that integrate traditional wisdom with scientific rigour can help make medicinal plant use safer, more effective and aligned with UHC goals.

## References

[1] WHO global report on traditional and complementary medicine. Geneva: World Health Organization; 2019 Available from: https://iris.who.int/handle/10665/312342 [cited 2025 Aug 6].

[2] Patwardhan B, Wieland LS, Kuruvilla S. WHO: a global boost for evidence-based traditional medicine. Nature. 2023 Aug;620(7976):950. 10.1038/d41586-023-02699-y 37644204

[3] Annex 1: Guidelines for selecting marker substances of herbal origin for quality control of herbal medicines. In: WHO Technical Report Series, No. 1003. Geneva: World Health Organization; 2017. Available from: https://www.who.int/docs/default-source/medicines/norms-and-standards/guidelines/quality-control/trs1003-annex1-marker-substances-herbal-medicine-quality-control.pdf?sfvrsn=f4ac0cca_0 [cited 2025 Aug 6].

[4] Patwardhan B, Chaturvedi S, Tillu G, Deshpande S, Hegde BM. Danish ban on Ashwagandha: truth, evidence, ethics, and regulations. J Ayurveda Integr Med. 2024 Jul-Aug;15(4):101028. 10.1016/j.jaim.2024.101028 38969606 PMC11403136

[5] WHO guidelines on safety monitoring of herbal medicines in pharmacovigilance systems. Geneva: World Health Organization; 2004. Available from: https://apps.who.int/iris/bitstream/handle/10665/43034/9241592214_eng.pdf [cited 2025 Aug 6].

[6] Nasim N, Sandeep IS, Mohanty S. Plant-derived natural products for drug discovery: current approaches and prospects. Nucleus (Calcutta). 2022;65(3):399–411. 10.1007/s13237-022-0 36276225 PMC9579558

[7] Kingsbury JM. The problem of poisonous plants. In: Kinghorn AD, editor. Toxic plants. New York: Columbia University Press; 1979 Dec 22. pp. 1–6.

[8] Wink M. Mode of action and toxicology of plant toxins and poisonous plants. Mitt Julius Kühn-Inst. 2009;421:93–112.

[9] Rasoanaivo P, Wright CW, Willcox ML, Gilbert B. Whole plant extracts versus single compounds for the treatment of malaria: synergy and positive interactions. Malar J. 2011 Mar 15;10(Suppl 1) Suppl 1:S4. 10.1186/1475-2875-10-S1-S4 21411015 PMC3059462

[10] Key technical issues of herbal medicines with reference to interaction with other medicines. Geneva: World Health Organization; 2021. Available from: https://www.who.int/publications/i/item/9789240019140 [cited 2025 Aug 6].

[11] Frontiers in Pharmacology. Real-world evidence of natural products, herbal medicines, and traditional medicine treatments. Volume II. Lausanne: Frontiers Media; 2024. Available from: https://www.frontiersin.org/research-topics/61054/real-world-evidence-of-natural-products-herbal-medicines-and-traditional-medicine-treatments-volume-ii [cited 2025 Aug 6].

